# Implementing online group model building to unravel complex geriatric problems, a methodological description

**DOI:** 10.1186/s12877-023-04110-x

**Published:** 2023-07-12

**Authors:** Oscar S Smeekes, Hanna C Willems, Ilse Blomberg, Etiënne A J A Rouwette, Bianca M Buurman

**Affiliations:** 1 Internal Medicine, section of Geriatric Medicine, Amsterdam UMC location University of Amsterdam, Meibergdreef 9, Amsterdam, 1105 AZ The Netherlands; 2 Medicine for Older People, Amsterdam UMC location Vrije Universiteit Amsterdam, Amsterdam Public Health Research Institute, De Boelelaan 117, Amsterdam, The Netherlands; 3 Department of Methodology, Nijmegen School of Management, Radboud University, Heyendaalseweg 141, Nijmegen, The Netherlands

**Keywords:** Implementation, Complex problems, Geriatrics, Online, Group model building, System dynamics

## Abstract

**Background:**

Group model building (GMB), is a qualitative focus group like study design from the field of system dynamics, that leads a group of topic experts (often key stakeholders of a problem), through a set of scripted activities to create a conceptual model of their shared view on this problems’ key contributing factors and their interactions. By offering a specific step wise approach to the complexity of a problem, GMB has provided better understanding and overview of complex problems across different scientific domains, in addition to traditional research methods. As the development of geriatric syndromes and organization of geriatric care are often complex issues that are difficult to research, understand and resolve, GMB might be a useful methodology to better address these issues. This study aimed to describe the methodology of online GMB using a geriatric case study.

**Methods:**

Four online GMB sessions were designed by two clinician researchers. A GMB methodology expert was consulted for optimal design. Scriptapedia scripts formed the core of the sessions. These scripts were adapted to the online format. Experts were recruited purposefully and included seven local health care professionals, one patient representative and one healthcare insurance data analyst. The outcome was a conceptual model of older adults’ emergency department visits, which was discussed in a separate article.

**Results:**

During implementation of these four sessions, the sessions were adjusted and two extra (non-scripted) sessions were added because defining unambiguous contributing factors to the geriatric case was challenging for the experts. Paraphrasing, categorizing, iterative plenary reflection, and reserving extra time were used to help experts overcome this challenge. All sessions were held in April and May 2021.

**Conclusion:**

This study shows that GMB can help unravel complex problems in geriatrics, both pathophysiological as organizational, by creating step wise overview of their key contributing factors and interactions. Furthermore, it shows that GMB can be used by clinicians, researchers and health policy makers to better understand complex geriatric problems. Moreover, this paper can help to overcome specific implementational challenges in the geriatric field.

**Supplementary Information:**

The online version contains supplementary material available at 10.1186/s12877-023-04110-x.

## Background

Group model building (GMB) is a participatory research method from the field of system dynamics that has provided new insight into the etiology of complex problems across different scientific domains [1]. In GMB, researchers facilitate specifically guided group discussion, with understanding of the problem studied, but without introducing their views on the problem [2, 3]. In these group discussions, that are conducted with topic experts (often key stakeholders of the problem), GMB captures their shared view on how the contributing factors of a complex problem interact by jointly constructing a model [2, 4–6]. This model summarizes the etiology of a complex problem. Traditional research methods do not provide this overview because they approach problems in a linear way, often identifying several interactions between factors without depicting their total coherence and impact [7]. GMB models also show how underlying mechanisms work and help to explore effective interventions [7]. Geriatric medicine is facing many complex problems, such as geriatric syndromes, dementia, emergency department (ED) visits, and the rehabilitation of patients with functional decline [8–12]. The complexity of these issues lies in the extensive interaction of many contributing factors from different scientific domains and the fact that these interactions are undefined [9, 10, 13–15]. GMB might help to address these complex problems.

The last decade has seen an increased application of GMB in healthcare, for example to address patient flow and workforce demand, and to understand syndromes like obesity, HIV/AIDS, and Alzheimer disease [9, 16–18]. Uleman et al. highlighted the potential of GMB in their comprehensible overview of Alzheimer disease and identified potentially important underlying mechanisms. However, despite these valuable results, studies have not investigated how to use GMB to address specific geriatric problems and examples of GMB in geriatric medicine are scarce [1, 9, 16, 17].

The COVID-19 pandemic has forced GMB studies to move online. This has advantages and disadvantages [19, 20]. Such as the advantage of logistics (planning and data collection in particular) and the disadvantage of more formal communication that potentially limits the sharing of ideas. Furthermore, facilitating online discussions requires a different set of skills and tools than in-person discussions do, but few studies have described these tools [19, 20]. A description of how to implement the GMB process online is needed. The implementation of GMB in the field of geriatric medicine has also not been well described [1, 9, 16, 17]. To address these gaps, this study aimed to describe the methodology of online GMB using a geriatric case study.

## Methods

### GMB compared with traditional research methods

GMB differs methodologically from traditional research methods in several ways, which make it useful for addressing complex problems. In comparison to in-depth interviews and the Delphi method, GMB involves group discussions, which give experts the opportunity to exchange views on the problem in person. This face-to-face interaction is essential for exploring a shared view together, especially if problems have an interdisciplinary character [5, 21]. Unlike focus groups, experts participate actively in forming a graphical depiction of the groups shared view and in doing so develop shared commitment [5, 21, 22]. In addition, GMB is facilitated by scripted activities, which optimize the use of different cognitive tasks, and step-by-step capture of the complexity of the problem in a model [3]. Main common features and differences between GMB and traditional research methods in researching the etiology of problems are described in Table 1.

**Table 1:** Main common features and differences between GMB and traditional research methods in researching the etiology of problems

	Group Model Building	Focus Group	Delphi method	Grounded Theory interviewing	Interpretative Phenomenological Analysis
Description Researchers selected and assembled…	A group of individuals jointly construct a model that reflects their shared view on a problems etiology through specifically scripted activities [5, 21].	A group of individuals discuss and comment on a problems etiology [23].	A group of individuals answer questionnaires on a problems etiology anonymously after which a summary of the groups’ results is presented and individuals have the possibility to revise their answers in reaction to these results in multiple rounds [24].	A single individual or a group of individuals is questioned individually on their view on a problems etiology [25].	A single individual or a group of individuals is asked individually to reconstruct their lived experience of a problem [26].
Aim(s) To better understand the problem by…	Breaking down the complexity of the problem, focusing on interaction between contributing factors, visualizing participants’ shared view on these interactions in a model and developing shared commitment.	Analyzing the exchange of views between participants.	Analyzing the often more consensus-based results or explanations of the final rounds.	Developing concepts during questioning and adjusting questioning along the way based on these developing concepts.	Analyzing individually reconstructions of lived experience.
Group discussion	Yes	Yes	No	No	No

### Types of models created by GMB

**Fig. 1:**
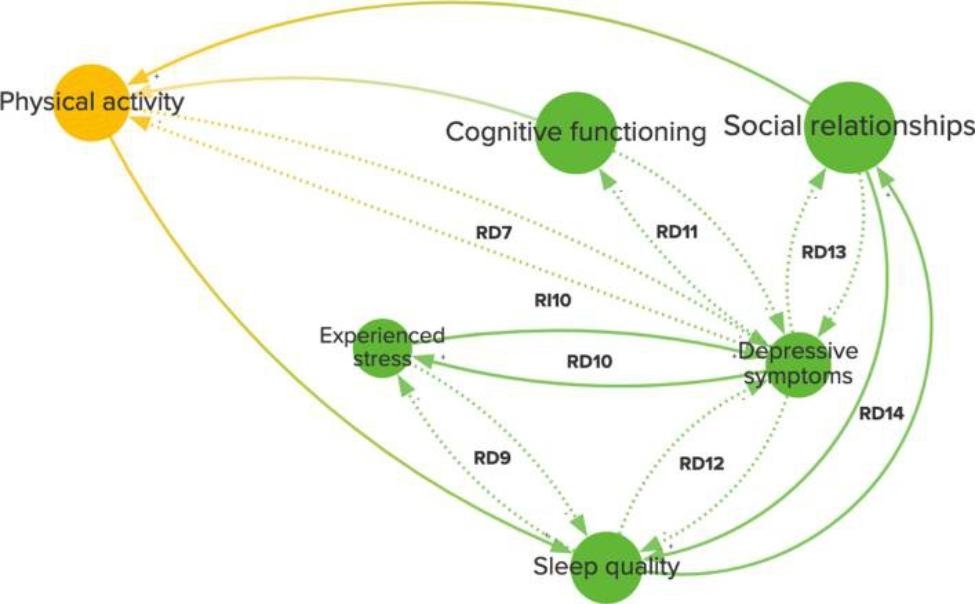
Example of a CLD on Alzheimer’s disease, from Uleman et al. [9]. This figure visualizes the role interactions between key causative factors can play in the etiology of a complex disease, such as Alzheimer’s. In this figure, physical activity and depressive symptoms, for example, are interconnected via feedback loop RD7. This feedback loop shows, that an increase in depressive symptoms will lead to a decrease of physical activity that can further increase depressive symptoms. In contrast to traditional research methods, the coherence of key causative factors and the feedback loops they are involved in tell us what effects need to be taken into account when exploring effective interventions. No changes to the figure were made. Copyright license: http://creativecommons.org/licenses/by/4.0/

Models created through GMB are system dynamic models [7, 27]. System dynamics is a simulation modelling approach addressing complex issues in various application domains and has been used extensively in healthcare [1, 16–18]. Different system dynamics models can be constructed, such as a causal loop diagram (CLD) or a stock and flow model (SFM). A CLD is a model type that provides overview of a complex problems most important causative mechanisms and is often the first step in system dynamics modeling of a problem [7, 28]. It does so by capturing only the key contributing variables, relations, and underlying mechanisms (feedback loops) of the problem [7, 29, 30]. In a CLD, variables are connected by arrows to illustrate a causal relation. When a closed circle of variables connected by arrows is formed, an underlying mechanism arises, informing the reader of the CLD of a hidden enhancing or balancing effect as the result of variable interaction [7]. An example of a CLD and the insights it can provide is given in Fig. 1. A SFM translates the same variables and relations into ‘stocks’ (elements that accumulate over time and can be measured) and ‘flows’ (elements that change over time)[28]. It brings a CLD closer to a computerized model, but can provide less overview of a problem’s underlying mechanisms and make capturing a problem more complex [28]. Once a CLD or SFM is constructed, the model is quantified and validated via literature and data [31]. This article describes the construction of a CLD that has been described in a previous case study [32].

### Case study

We conducted a case study between February and May 2021 to better understand why people older than 65 years of age visit the ED in Amsterdam at a population level without excluding any subcategories of older adults (see Appendix 1). The results of this case study have been published separately [32]. In the present study, we used an online GMB research design to capture the views of an interdisciplinary expert group on the interactions of most important contributing factors to these older adults’ ED visits as a population in a CLD. The CLD depicted the year 2019 to exclude the effects of the COVID-19 pandemic. These sessions were done online because of COVID-19 restrictions. OS and IB both designed as well as facilitated the GMB sessions, HW facilitated the GMB sessions and clarified output, and ER gave expert advice on design. Further information on the researchers’ backgrounds can be found in the section on authors’ information. All methods were carried out in accordance with relevant guidelines and regulations.

### GMB protocol

#### Sessions, scripts, and adaptations

**Fig. 2:**
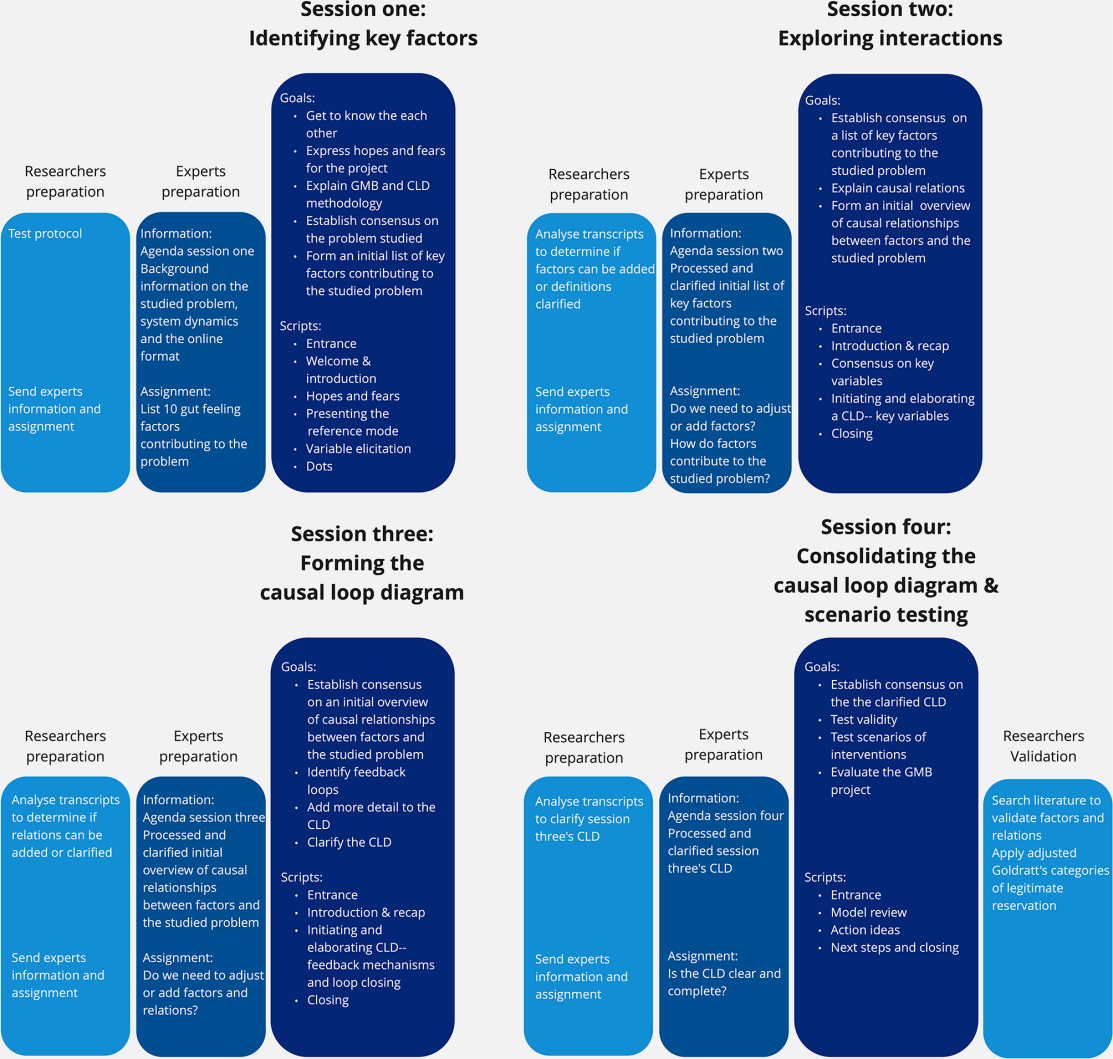
Overview of GMB design, including session themes, scripts, goals, and preparation

To design a GMB protocol that fitted to the case study, we consulted the literature [4, 5, 30, 33, 34], Scriptapedia (an open access online book containing guidelines and scripts for evidence-based GMB [35]), and our own expertise. The protocol was designed in February, March, and April 2021 and the sessions, goals, scripts, and preparation are summarized in Fig. 2. All sessions and scripts were adapted for online use and these adaptations are included in Table 2.

**Table 2:** GMB preparation assignments and scripts, including name, description, goal, motivation for selection, and adaptation

Name	Description	Goals	Motivation for selection	Adaptation
‘Expert preparation’ (A)^1^ Session one	Background information on older persons’ ED visits in Amsterdam, system dynamics (GMB and CLD), and the online format are sent to the experts by email before the first session. Experts are asked to write down their ten most important factors contributing to the problem based on their gut feeling. This assignment is derived from Vermaak (18). The agenda and an explanation of the session focus are also included.	To enhance in-session performance by stimulating experts to prepare themselves for the first session. To have a list of factors on paper before the start of the GMB.	See goals.	-
‘Expert preparation’ (A) Session two	The processed and clarified initial list of key factors contributing to the studied problem are sent to the experts by email before the second session. Experts are asked to reflect on the following questions: Do we need to adjust or add factors? How do factors contribute to the studied problem? The agenda and explanation of session focus are also included.	To enhance in-session performance by stimulating experts to contribute factors to the studied problem. To develop new insights to improve the CLD.	See goals.	-
‘Expert preparation’ (A) Session three	The processed and clarified initial overview of causal relationships between factors and the studied problem are sent to the experts by email before the third session. Experts are asked to reflect on the following questions: Do we need to adjust or add relations? The agenda and explanation of the session focus are also included.	To enhance in-session performance by stimulating experts to contribute factors to the studied problem. To develop new insights that improve the CLD.	See goals.	-
‘Expert preparation’ (A) Session four	The processed and clarified CLD are sent to the experts by email before the fourth session. Experts are asked to reflect on the following questions: Is the CLD clear and complete? The agenda and explanation of the session focus are also included.	To enhance in-session performance by stimulating experts to contribute factors to the studied problem. To develop new insights that improve the CLD.	See goals.	-
‘Entrance’ (A) All sessions	Before starting every meeting, the facilitators ask experts to check for stable internet connection, turn cameras on, mute when not speaking, raise their hand if they have a question, and wait their turn to speak.	To check and set rules for optimal communication.	See goals.	-
‘Welcome and introduction’ (A) Session one	The facilitators welcome and thank experts for their participation. They share the enthusiastic reactions received from experts during the recruitment. They present an overview of session themes and agenda for the first session.	To explicate researchers’ enthusiasm for the project as well as to share experts’ enthusiasm. Thereby aiming to connect experts by shared enthusiasm. To create a dosed overview of the GMB process for experts.	To have a good basis for the GMB goals in the first session.	-
‘Hopes and fears’ (S)^2^ Session one	The facilitators ask experts to write down expectations for the GMB session or project on post it’s. They are then compiled and read out loud before they are added to a board.	To elicit and establish expectations as a group.	By addressing expert and facilitator expectations for the process before starting with the model building, the researchers aim to connect with the experts, make them connect as a group, and boost motivation.	The original script is adapted by asking researchers first and experts second in a round robin fashion to introduce themselves, describe their expertise, and name one hope and one fear. The researchers aim to lower the threshold for taking the floor by starting with themselves. Hopes and fears are directly placed on the Miro board, but shown to the experts only after all experts shared, to keep focus on the person instead of the Miro board. All hopes and fears are explicated by the facilitators. The original time frame was reduced to direct plenary discussion of one hope and one fear.
‘Presenting the reference mode’ (S) Session one	The facilitators present the problem selected. The experts establish the problem to be studied.	To reach consensus on the dynamic problem.	The model construction starts by finding consensus on the problem and its behavior.	The script is adapted by adding a short PowerPoint presentation recapping the problem. The focus is on finding consensus on a clear definition of the problem. Less focus is on its dynamic behavior over time, because this attributes to the complexity for experts. No graphs over time are used. PowerPoint slides are shared in Zoom and experts vote on the studied problem by raising their hand if they agree. If a hand is not raised, an explanation is asked for. If new suggestions are made for the studied problem, the experts vote until consensus is reached. Consensus on the problem elicitation is expected quickly, so only five minutes are reserved for this script.
‘Variable elicitation’ (S) Session one	The facilitators ask experts to write down as many key factors contributing to the problem and vice versa. They are then added to a board.	To facilitate divergent group discussion about factors contributing to the studied problem.	The most logical step after reaching consensus on the problem is using a script that asks experts to name key contributing factors to the studied problem, thereby providing a basis for a CLD.	This script is adapted using the preparation assignment for session one as initial input. This gives experts have extra time and resources to come up with a clearly described list of possible factors that are ready to be shared directly after the script starts. The facilitators ask experts to name the factors one by one in round robin fashion. Experts are asked not to name factors that have already been named. Factors are shown directly in Miro. Facilitators and experts are asked to clarify the factors.
‘Dots’ (S) Session one	The facilitators ask experts to divide three or five votes over factors and place them on a board.	To highlight the most important factors for the experts.	This script is selected to give the experts a first impression on a selection of most important factors for the group as a whole before starting to describe relationships.	Experts are asked in round robin fashion to assign five dots in random distribution to the most important factors. These dots are added to the Miro board directly.
‘Introduction and recap’ Sessions two, three, and four (A)	The facilitators ask experts for their reflections on the preparation information and assignments.	To check for unclarities, new ideas, and agreement on the CLD.	This allows the facilitators to check and address any unclarities about the CLD. This gives experts a jump start for the next script.	-
‘Initiating and elaborating a CLD’ (S) Sessions two and three	The facilitators add identified contributing factors to the studied problem one by one to a board. Then the causal relationship with the problem is described.	To form an initial CLD.	This script was chosen because the researchers believed it provides an overview of the problem, which was one of their main goals.	An individual exercise is added to the original script because it is hypothesized that a lot of factors will be identified and experts will find it difficult to describe relationships in a plenary ad hoc fashion. In the exercise, each expert is assigned a factor and has to describe the relationship to the problem and all other mentioned factors. After this, the results are discussed plenary one by one and put on the online whiteboard. The time frame is widened because of the comprehensiveness of the number of relationships expected.
Closing (A) Sessions one, two and three	The facilitators close the session by summarizing the progress made in the CLD construction and explaining the focus of the following session.	To motivate experts on progress and outline the following step in the CLD construction.	To close sessions effectively.	-
Model review (S) Session four	The facilitators give a plenary demonstration of the CLD formed.	Experts reflect on adequacy of the CLD formed and the need for alterations.	The script is selected because it clearly demonstrates, stresses, and reflects on the CLD.	Because of its good fit, no adjustments are made except for the online format. The adjustments include screen sharing of the CLD in Miro, capturing feedback from experts in Miro, and reserving more time for feedback.
Action ideas (S) Session four	The facilitators ask experts to come up with as many ways to improve the system modeled and share their most important one. Experts’ suggestions are placed in a quadrant illustrating difficulty to achieve and size of effect.	To prioritize actions after a model has been developed.	The script was selected because it lets experts think about solutions from a system dynamics effect viewpoint.	Because of its good fit, no adjustments are made except for the online format. The adjustments include screen sharing of the CLD and quadrant in Miro, capturing feedback from experts in Miro, and reserving more time for feedback.
Next steps and closing (S) Session four	The facilitators present the next steps that will be taken after the GMB session.	To inform the experts on follow up.	This script is chosen because it provides clear closure.	Because a detailed agenda of the study is shared with the experts repeatedly, less time is reserved for this script.

^*1*^ *Activities marked with (S) are Scriptapedia-derived scripts and were adapted by the researchers to fit the studied problem, session goals and online format.* ^*2*^ *Activities marked with (A) were designed by the researchers to enhance the CLD building process and are therefore additive*

The GMB protocol was designed as four 1.5-hour sessions conducted a few days apart. This was done for several reasons. First, 1.5-hour sessions allowed the experts to focus on a limited number of activities and thereby on each step of the model build. Second, conducting sessions a few days apart allowed us to give the experts information and assignments to prepare for the sessions, thereby enhancing session performance. Third, time between sessions allowed experts to consolidate the knowledge they gained during sessions and to develop new perspectives [36]. Four, several shorter sessions made it easier for experts to fit sessions into their schedule. Last, time between sessions allowed us to process the session output and improve its visualization before the next session.

The sessions addressed the following themes: identifying key factors (session one), exploring interactions (session two), forming the CLD (session three), consolidating the CLD, and testing scenarios (session four).

Sessions were structured using GMB activities called scripts. Scripts were compiled on Scriptapedia and included 38 scripts [cited 1st of June 2022] [35]. The scripts were divided into three categories: established (n = 21), promising (n = 12), or under development (n = 5). These scripts have been validated by multiple independent teams and produce consistent results (26). Scripts were also divided by the type of cognitive tasks they entail; these were introductory/presentation (designed to educate or update experts), divergent (designed to produce different ideas and interpretations), convergent (designed to cluster and categorize ideas and interpretations), and concluding/evaluative (designed to rank and choose between options and ideas).

Only established scripts from Scriptapedia were selected for the sessions. These scripts included ‘hopes and fears’, ‘presenting the reference mode’, ‘variable elicitation’, ‘dots’, ‘initiating and elaborating a CLD’, ‘model review’, ‘action ideas’, and ‘next steps and closing’[35]. Additional scripts were designed by the researchers to enhance session performance. These included ‘entrance’, ‘welcome and introduction’, ‘introduction and recap’, and ‘closing’ and checked the experts’ understanding of the content, methodology, and medium (Zoom and Miro) as well as their perspectives on the CLD.

Adapting the GMB protocol to an online format included altering the organization, visualization, and communication of the scripts. We used a recent article on conducting GMB online as a guideline [19]. For communication, Zoom (video communication [37]), Miro (an online whiteboard [38]), and email were used. To maintain focus on the model under construction, experts were given ‘read-only access’ to Miro for reflection and were only allowed to speak after using the raise-hand function in Zoom (see Tables 2 and Appendix 2 for more details). To minimize the chance of potentially limiting the sharing of ideas by experts and bias in their response as a result of the online format, we aimed to create a safe, non-judgmental, informal setting (however with clear communication protocol) and gave every expert a chance to share ideas or interrupt using the raise hand function. Experts were given preparative information and assignments before each session to stimulate thoughts on the process and enhance session performance (see Table 2).

#### Roles and facilitation manuals

Sessions were led by three facilitators (OS, IB, HW, see the section on authors’ information for their background). To facilitate the scripted GMB process, researchers take on different roles [4]. A minimum of five essential roles are needed: facilitator, modeler/reflector, process coach, recorder, and gatekeeper [4]. In our study, OS (facilitator one) focused on group facilitation, knowledge elicitation, and initial drafts of the structure. IB (facilitator two) fulfilled the recorder role by collecting data and conceptualizing the system. OS and IB fulfilled the modeler/reflector role. HW (facilitator three) took on the roles of gate keeper and process coach. HW evaluated the group dynamics and helped to frame the problems discussed in the first session. In later sessions, HW reflected on group and facilitator team dynamics. We used facilitation manuals to design and implement the GMB process. These included an in-depth description of the sessions’ objectives, roles, scripts, and agendas [35] and are presented in Appendix 2.

#### Expert selection

We purposefully recruited experts based on a predetermined essential profiles list in order to achieve the research aim, account for optimal online discussion group size as suggested by ER and Wilkerson et al., as well as to reduce the risk of selection bias. In GMB, expert selection involves multiple experts that are central to a topic and experts are selected using different methods [6]. Expert profiles included key stakeholders of the older adults’ patient journey leading to ED visits in Amsterdam, who were seen as an expert by colleagues and had at least five years of job experience. Nine essential expert profiles were identified for our case study, including seven local health care professionals (district nurse, ED physician, general practitioner, geriatrician, geriatrics physician, nurse specialist geriatrics, nurse transfer coordinator), one patient representative and one healthcare insurance data analyst. Nine experts were recruited and formed a fixed participant group during the GMB study. Expert selection was described in detail previously [32]. A summary of their characteristics can be found in appendix 3.

### Data collection, data analyses, and model validation

Data were collected by video recording the sessions and capturing expert discussion on the online whiteboard. Video records were transcribed verbatim, anonymized, and checked for accuracy. As in line with Scriptapedia guidelines [35], data were analyzed and validated as part of the scripts and between sessions. All output, analyses and validations were discussed with the expert group. More details are presented in Table 2. and Fig. 2. Validation of the model was previously described [32].

## Results

### Session implementation and adjustments

**Fig. 3:**
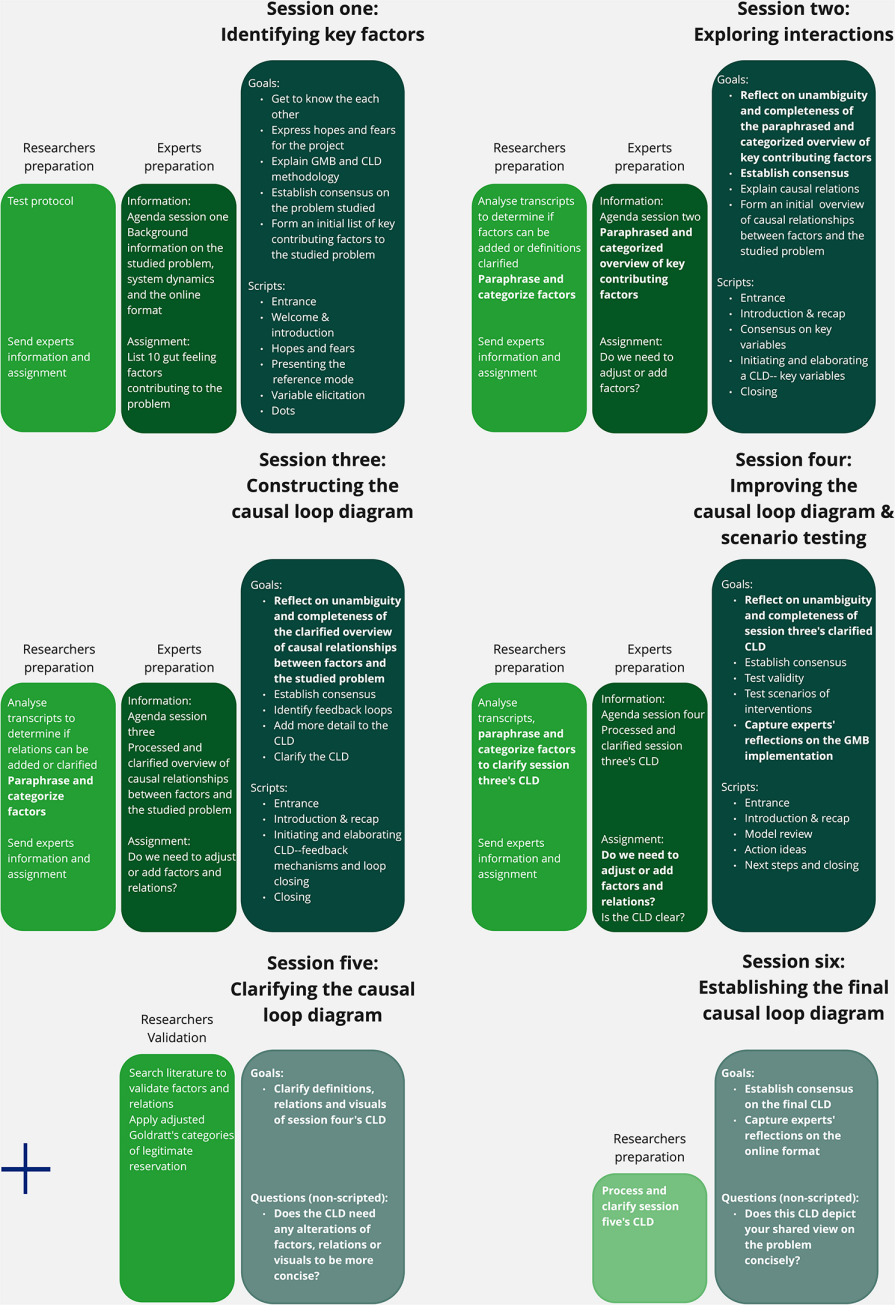
Overview of GMB implementation, including session themes, scripts, goals, and preparation

All sessions were held in April and May 2021 and produced a CLD that visualized the combined expert view on why older people visit the ED in Amsterdam. Sessions one to four lasted 1.5 h. Sessions were adjusted because experts found it challenging to define unambiguous contributing factors. Two non-scripted sessions were added to clarify the CLD and establish consensus on a final version. The final protocol is shown in Fig. 3 and all script adjustments are presented in Table 3. The experts’ reflections on implementation are summarized in Appendix 4.

**Table 3:** Adjusted GMB preparation assignments and scripts

Name of script	Adjustments
‘Expert preparation’ (A) Session two	The information experts receive is paraphrased and categorized in an overview of key contributing factors. The assignment includes the question: Do we need to adjust or add factors? The question How do factors contribute to the studied problem? is excluded.
‘Expert preparation’ (A) Session three	The information experts receive is paraphrased and categorized in an overview of causal relationships between factors and the studied problem. The assignment includes the question: Do we need to adjust or add factors and relations? The question about the factors is additional.
‘Expert preparation’ (A) Session four	The information experts receive is paraphrased and categorized in the CLD clarified in session three. The assignment includes the questions: Do we need to adjust or add factors and relations? Is the CLD clear? The question about the factors is additional.
‘Introduction and recap’ Sessions two, three, and four (A)	The primary focus of these scripts was shifted to plenary reflection on factor definitions.
‘Initiating and elaborating a CLD’ (S) Sessions two and three	Extra time was scheduled for this script. Extra time was saved by processing some experts’ suggestions after the session ended.
Model review (S) Session four	Extra time was scheduled for this script and it was merged with the ‘introduction and recap’ script.

*Scripts were adjusted to give the experts the tools they needed to overcome the challenge of defining contributing factors.*

Three adjustments were made to help experts better define contributing factors. First, all facilitators used their own expertise to paraphrase factors in an unambiguous way during the sessions and these paraphrases were approved by the experts. These discussions resulted in unambiguous definitions for most factors. Second, the modelers categorized the suggested factors using the Miro whiteboard and these categories were approved by the experts. This facilitated group discussion on ambiguity and contributed to unambiguous definitions for several factors. It also helped the researchers to organize the CLD in a clear way. Third, the facilitators planned iterative plenary reflection on suggested factors at the beginning of each session, which also sharpened the definitions.

Extra time was scheduled for scripts that defined contributing factors, including ‘initiating and elaborating on a CLD’ and ‘model review’. Time was saved by processing some of the suggestions made by experts in the ‘initiating and elaborating a CLD’ script after the session.

A fifth non-scripted facultative session was added to discuss the clarity of the CLD. In GMB, CLD clarity is often checked by the modelers [1, 16, 35]. We invited the experts to join the check because defining contributing factors was challenging. The ED physician, geriatric nurse, and district nurse participated. This clarified the three factors and added two relations.

A sixth non-scripted mandatory session was also added to establish consensus among the experts on the final CLD. This final presentation of the CLD is often not considered part of the GMB [1, 16, 35]. Because definitions were challenging, a mandatory session was organized with all experts to check if the CLD clearly depicted the experts’ views. Experts established consensus on the CLD formed in session five (see Fig. 3 and Appendix 2 for more details).

## Discussion

This study describes a methodological GMB approach in geriatric medicine. It was challenging to implement four qualitative online GMB sessions on why older adults visit the ED. We adjusted the protocol to help the experts better define contributing factors. These adjustments included reserving extra time for discussion, paraphrasing definitions, categorizing definitions, and reflecting with experts on suggested factor definitions. Communication was promoted by giving every expert the chance to speak combined with a clear communication protocol. Six sessions were held altogether, which resulted in a clear CLD.

To the best of our knowledge, no other study has reported difficulty using GMB methodology to define factors contributing to why older adults visit the ED. We believe that the challenges we observed were caused by the characteristics of the ED visits, such as frailty, which are poorly defined, hard to measure, and the result of multiple contributing factors [12, 39, 40]. The CLD formed in this study underlines this hypothesis [32]. Traditional GMB methodology [4, 5, 30, 33–35] did not provide the tools to unambiguously define these characteristics so we developed alternative ways to use the methodology. ED visits by older adults have similar characteristics to many problems in geriatric medicine, such as geriatric syndromes and the rehabilitation potential of patients with functional decline [8, 11]. Therefore, defining contributing factors for these many problems is expected to be challenging.

Paraphrasing, categorizing, iterative plenary reflection and reserving extra time for defining contributing factors helped our experts to better define these factors. These tools clarify and organize the conceptualization of contributing factors [36, 41, 42]. In line with these findings, the Scriptapedia guidelines describe paraphrasing as an important tool in the GMB process, but the skills required for paraphrasing are not described [35]. In this study, we found that a good clinical background in geriatric medicine was essential for paraphrasing definitions effectively. To our knowledge, categorization has not been reported as a tool to help experts define contributing factors in GMB studies. We found that extensive knowledge on the problem under discussion was essential for effective categorization. Reserving extra time and plenary reflection have often been used in GMB, but not for defining contributing factors. In summary, we advise using these four tools when applying GMB in geriatric medicine and having at least one researcher on the team with a clinical background in geriatrics and extensive knowledge on the problem being studied.

Scripts have to be adapted for online use. Wilkerson et al. have produced practical guidelines for adjusting scripts to an online format and we followed these guidelines when designing the present study [19]. We made a few additions to Wilkerson’s guidelines, such as giving every expert a chance to speak. This, combined with a clear communication protocol, improved the discussions and we recommend these measures in future online GMB studies.

A strength of this study is the detail of process description. The rationale of GMB studies is often limited to script adaptation [1], which means valuable insights into effective implementation are missed. An additional strength is that this study was conducted online effectively and therefore represents an example of the logistical benefits of online GMB.

### Limitations

A shortcoming of this study is that no GMB methodology expert facilitated the sessions. We addressed this by consulting a methodology expert (ER) in advance about the study design and by using our own expertise. Furthermore, paraphrasing and categorizing the definitions of contributing factors may have introduced bias. We minimized this risk by asking the experts if they approved these changes. Finally, by including only one expert from a different scientific discipline (healthcare insurance data analyst/economics) we may have introduced a bio-medical bias on response. However, healthcare professionals working in geriatrics, diagnose and treat psychosocial problems every day as a part of their profession and see the effects of laws as well as financial problems contribute to older persons’ ED visits. Furthermore, these problems were extensively visualized in the CLD.

This study contributes to both research and clinical practice by offering an example of how online GMB can be used to better understand complex problems in geriatrics. This example can help both clinician, researcher as policy maker to use GMB for addressing complex geriatric problems. These complex problems can be biopsychosocial, organizational or intertwined. In order to secure a holistic approach to these problems, representativeness of different scientific disciplines, such as sociology, anthropology, psychology, economics, mathematics, law, philosophy etc., in GMB expert selection should be taken into consideration. More online GMB studies are needed in geriatric medicine to validate the challenges and possible solutions we have identified in this methodological approach.

## Conclusions

In sum, we have described the methodological approach for applying GMB to unravel complex problems in geriatric medicine. We also tested alternative ways of using the methodology to help experts overcome the challenge of defining contributing factors in a geriatric case study. These ways included paraphrasing and categorizing the definitions, offering plenary reflection, and reserving extra time for defining contributing factors. Giving every expert the chance to speak combined with a clear communication protocol also promoted orderly communication. Since the characteristics of this geriatric case study are similar to many geriatric problems, the insights from this study may improve the application of GMB in geriatric medicine.

## Electronic supplementary material

Below is the link to the electronic supplementary material.

Supplementary Material 1

## Data Availability

The majority of data generated and analyzed during the current study are publicly available and included in either the manuscript or additional file. Restrictions apply to the availability of the transcripts, which were used under approval of the experts for the current study, and so are not publicly available. Transcripts are however available from the authors upon reasonable request and with the permission of the experts. Transcripts are in Dutch.

## Declarations

### Authors’ information

OS is an MD PhD student on the DOLCE VITA project, researching the etiology of older adults’ ED visits, and a fifth-year internal medicine resident specializing in geriatric medicine. He has extensive experience in clinically assessing older adults at the ED in Amsterdam, both at a city-center hospital and the academic center. Furthermore, he has extensive experience in moderating group discussion and has conducted focus groups in the past. IB is a medical student in her last year of training and is writing her master’s thesis on the case study under supervision of OS, HW and BB. She has experience in clinically assessing older adults at the ED of a city-center hospital in Amsterdam and moderating group discussion. HW is an MD PhD internist-geriatrician and is head of the department of geriatric medicine at Amsterdam UMC. She has extensive expertise in geriatric medicine and in conducting focus groups. She is a co-supervisor of OS. ER is professor at the Methodology Department of the Nijmegen School of Management, Radboud University (the Netherlands). He is specialized in GMB methodology. BB is professor at the department of geriatric medicine at Amsterdam UMC and is chair of the Dutch society for Nurses. She is specialized in Acute geriatric care. She has extensive expertise in the acute geriatric research field. She is a supervisor of OS.

## List of Abbreviations

GMB: Group model building
ED: Emergency department
CLD: Causal loop diagram
SFM: Stock and flow model
