# Supplementary material for: Implementing online group model building to unravel complex geriatric problems, a methodological description
Source: BMC Geriatr. 2023 Jul 12;23:431. doi: 10.1186/s12877-023-04110-x (PMC10339532; doi:10.1186/s12877-023-04110-x)
Supplement: Supplementary file 1 — Supplementary Material 1 [file 12877_2023_4110_MOESM1_ESM.docx]

**Appendices 1-4 Implementing online group model building to unravel complex geriatric problems: a methodological description, including description of study setting, facilitation manual, experts’ characteristics and reflections on implementation**

1. Definition of the Amsterdam area and setting
2. Detailed facilitation manuals
3. Experts’ characteristics
4. Experts’ reflections on implementation
5. **Definition of the Amsterdam area and setting**

This research was nested within the Data driven Optimization for a Vital elderly care system in the Netherlands (DOLCE VITA) project. The project aims to elucidate acute care for older adults in the Netherlands, particularly in Amsterdam, and to improve future policy-making using model development. The program involved two PhDs with a medical and mathematical background, and multiple clinicians and researchers from medical and mathematical fields. In the DOLCE VITA project, Amsterdam included Amstelveen and Diemen and this geographical area has nearly one million inhabitants with a diverse demography and a range of healthcare providers, including one tertiary hospital (two locations), two larger city hospitals (one with two locations), and one smaller teaching hospital. This GMB study was conducted in the same geographical region as the DOLCE VITA research project.

1. **GMB facilitation manuals**

*Allocation of responsibilities to the researchers*

| Name | Responsibilities |
| --- | --- |
| MD O.S. Smeekes (OS) | - GMB design - Recruitment of experts - Logistics and planning - Development of preparation materials and facilitation manual - Preparation of introductory presentations and scripts - GMB facilitation: facilitator one - Data collection and analysis - Validation |
| I.G. Blomberg (IB) | - GMB design - Recruitment of experts - Logistics, planning, and contact with experts - Development of preparation materials and facilitation manual - Preparation of Zoom and Miro - GMB facilitation: facilitator two - Data collection, transcription, and analysis - Validation |
| Dr. MD H.C. Willems (HW) | - Support of the recruitment strategy - Feedback during preparation phase - GMB facilitation: facilitator three - Reflection on the GMB facilitation and process |

*Allocation of GMB roles (as described by Scriptapedia, cited 1^st^ of June 2022* [1]*) to the researchers*

| Roles | Description specified to the case study | Name |
| --- | --- | --- |
| (Community) Facilitator | - Directs the GMB process - Provides the right ‘’facilitative attitude’’ - Facilitates experts in knowledge elicitation using geriatric background - Starts and closes the sessions - Paraphrases and categorizes session output | OS |
| Modeler/reflector | - Builds the causal loop diagram, models, and simulations - Reflects with experts on progress and challenges - Facilitates experts in knowledge elicitation using geriatric background - Paraphrases and categorizes session output | OS and IB |
| Recorder | - Takes notes and facilitates recordings - Keeps track of the time | IB |
| Gatekeeper | - Ensures that the project is meeting the goals and provides feedback on progress and results - Supports in framing the problem - Identifies appropriate experts | HW |
| Process coach | - Observes the group’s progress - Gives feedback to the modelers about group dynamics and progress | HW |

*GMB sessions in detail*

This section provides details of the six GMB sessions, including the objectives, activities, anticipated outputs, detailed agendas, and team roles.

**Session one**

*Objectives*

Explicit To identify which factors contribute to older persons (65+) visiting the ED in Amsterdam

To establish consensus on the studied problem

Implicit To introduce the facilitators to the experts and each other

To create understanding of and familiarity with the GMB process

*Summary of activities*

| Activity/script | Description | Anticipated output |
| --- | --- | --- |
| Introduction | Welcome experts  Introduction to the GMB sessions | Welcoming environment |
| Hopes and fears (variation)  *Divergent script* | Experts and core facilitation team introduce themselves and share their motivation and expectations (hopes and fears) for the GMB sessions | Icebreaker  List of expectations |
| Reference mode (variation)  *Presentation script* | Facilitators check whether the group has consensus on the problem articulation and has understood the preparation documents | Consensus on the articulated problem |
| Variable elicitation  *Divergent script* | Experts generate an initial set of key factors contributing to the articulated problem | List of key factors |
| Dots  *Evaluation script* | Experts individually vote which factors they consider to be most important | Prioritized choices |

*Detailed agenda*

| Time | Min | Activity | Facilitator one  *Facilitator, modeler/reflector* | Facilitator two  *Recorder, modeler/reflector* | Facilitator three  *Gatekeeper, process coach* |
| --- | --- | --- | --- | --- | --- |
| 15:50 - 16:00 | 10 | Entrance | Welcomes experts | *[Zoom, gallery view]*  Checks technical setup experts  Starts recording | Welcomes experts |
| 16:00 - 16:05 | 5 | Welcome and introduction | Opening remarks:  Introduces project and first session | *[Zoom, gallery view]*  Assists experts with technical issues | Observes group dynamics and supports facilitation |
| 16:05 - 16:20 | 15 | Hopes and fears | Introduces script by asking experts to introduce themselves and share a hope and a fear, starting with the facilitation team | *[Zoom, gallery view]*  Captures hopes and fears on Miro | Observes group dynamics and supports facilitation |
| 16:20 - 16:25 | 5 | Reflection on hopes and fears | Supports facilitation | *[Screen share, Miro]*  Explains Miro | Shares reflections on hopes and fears |
| 16:25 - 16:30 | 5 | Reference mode | Gives brief recap of preparation materials and problem articulation | *[Screen share, introduction presentation]* | Observes group dynamics and supports facilitation |
| 16:30 - 16:20 | 50 | Variable elicitation | Asks experts to share key factors one by one  Factors may only be called once  Continues until the group is satisfied | *[Screen share, Miro]*  Captures key factors on Miro board    Clarifies definitions | Observes group dynamics and supports facilitation |
| 17:20 - 17:27 | 7 | Dots | Introduces the script and gives each expert “5 votes” to send by chat | *[Screen share, Miro]*  Copies dots from chat to Miro | Observes group dynamics and supports facilitation |
| 17:27 - 17:30 | 3 | Closing | Closing remarks: outlines plan for session two | *[Zoom, gallery view]* | Shares reflections on session 1 |

**Session Two**

*Objectives*

Explicit         To reflect on unambiguity and completeness of the paraphrased and categorized overview of key contributing factors

To establish consensus on key factors

To form an initial overview of causal relationships between factors and the studied problem based on expert’s thoughts and stories

Implicit Experts understand the concepts of causal relationships and start to see a system regarding why older people visit the ED

*Summary of activities*

| Activity | Description | Anticipated output |
| --- | --- | --- |
| Introduction and recap | Facilitators ask experts for plenary reflection on the paraphrased and categorized results from the *Variable elicitation* and *Dots* scripts | Supplemented list of factors and dots |
| Consensus on key factors  *Evaluation script* | Group establishes consensus on 8 to 10 key factors | Consensus on prioritized factors |
| Initiating and elaborating a CLD  *Convergent script* | Facilitators help the group initiate CLD construction starting with the problem factor and adding key factors | Experts understand the concepts of causal relations in system dynamics  Initial overview of causal relationships between factors and the studied problem |

*Detailed agenda*

| Time | Min | Activity | Facilitator one  *Facilitator, modeler/reflector* | Facilitator two  *Recorder, modeler/reflector* | Facilitator three  *Gatekeeper, process coach* |
| --- | --- | --- | --- | --- | --- |
| 19:20 - 19:30 | 10 | Entrance | Welcomes experts | *[Zoom, gallery view]*  Fixes technical issues  Start recording | Welcomes experts |
| 19:30 - 19:45 | 15 | Introduction and recap | Opening remarks: Welcome and brief recap of session 1  Facilitators ask experts for plenary reflection on the paraphrased and categorized factors | *[Zoom, gallery view]*  *[Screen share, Miro]*  Show paraphrased and categorized factors  Clarifies unclear definitions | Observes group dynamics  Observes group dynamics |
| 19:45 - 20:00 | 15 | Consensus on key factors | Explores consensus on key factors by all experts | *[Screen share, Miro]*  Shows categorized key factors and captures consensus on Miro | Observes group dynamics |
| 20:00 - 20:05 | 5 | Introduction to script | Introduces causal relations thinking by giving a simple example | *[Screen share, Miro]*  Assists F1 by visually illustrating an example on Miro | Makes sure everyone understands the introduction |
| 20:05 - 20:55 | 50 | Initiating and elaborating a CLD | Assists the group to initiate construction of an initial overview of causal relationships between factors and the studied problem starting with problem factor in the middle | *[Screen share, Miro]* Captures feedback loops with green (+) and red (-) lines on Miro | Observes group dynamics |
| 20:55 - 21:00 | 5 | Closing | Closing remarks: outlines plan for session three | *[Zoom, gallery view]* | Observes group dynamics |

**Session Three**

*Objectives*

Explicit         To reflect on unambiguity and completeness of clarified overview of causal relationships between factors and the studied problem

To establish consensus on the overview

To identify feedback loops

To clarify and add more detail to the CLD

Implicit        To clarify the complexity of the problem

*Summary of activities*

| Activity | Description | Anticipated output |
| --- | --- | --- |
| Introduction and recap | Facilitators ask experts for plenary reflection on the clarified overview of causal relationships between factors and the studied problem | Supplemented overview  Consensus on the overview |
| Initiating and elaborating a CLD  *Convergent script* | Facilitators assist the group to continue with CLD construction by discussing the feedback mechanisms and closing the loops | Clarified and more detailed CLD  Consensus on definitions |

*Detailed agenda*

| Time | Min | Activity | Facilitator one  *Facilitator, modeler/reflector* | Facilitator two  *Recorder, modeler/reflector* |
| --- | --- | --- | --- | --- |
| 15:55 -  16:00 | 5 | Entrance | Welcomes experts | *[Zoom, gallery view]*  Fixes technical issues  Starts recording |
| 16:00 -  16:15 | 15 | Introduction and recap | Welcome and recap of session two.  Facilitators ask experts for plenary reflection on the clarified overview | *[Zoom, gallery view]*  *[Screen share, Miro]*  Shows overview of key factors and clarified overview  Captures input |
| 16:15 -  16:20 | 5 | Initiating and elaborating a CLD | Explains individual task for experts  Each expert is assigned to a specific factor | *[Screen share, Miro]*  Shows overview with which expert works on which key factor  Shows exercise example |
| 16:20 -  16:30 | 10 | Initiating and elaborating a CLD  *Individual task* | Each expert draws all connections from the assigned key factor to the other factors and problem factor | *[Screen share, Miro]*  Shows updated CLD |
| 16:30 -  17:00 | 30 | Initiating and elaborating a CLD  *Group task* | Asks each expert to share new drawn connections and discuss this with group | *[Screen share, Miro]*  Shows updated CLD and draws new connections. Captures input on Miro |
| 17:00 -  17:25 | 20 | Initiating and elaborating a CLD  *Group task* | Addresses each key factor and asks experts one by one to name influencing factors not mentioned before | *[Screen share, Miro]*  Captures input on Miro |
| 17:25 -  17:30 | 5 | Closing | Closing remarks: outlines plan for session four | *[Zoom, gallery view]* |

**Session Four**

*Objectives*

Explicit        To reflect on unambiguity and completeness of the CLD clarified in session three

To establish consensus on the CLD

To test the CLD’s validity

To test scenarios of interventions

        To capture the experts’ reflections on GMB implementation

Implicit        Experts have high energy and enthusiasm in finding potential solutions

To create shared understanding of interventions and why some ideas might be effective and others are not

*Summary of activities*

| Activity | Description | Anticipated output |
| --- | --- | --- |
| Introduction and recap + model review  *Convergent script* | Facilitators ask experts for plenary reflection on the CLD clarified in session three after a walk through and validity test | Consensus on new version of the CLD |
| Action ideas  *Divergent script* | Experts share potential solutions that affect the CLD. The group discusses the workability and priority of the ideas and how they connect with the CLD. | Prioritized overview of potential actions |
| Next steps and closing  *Convergent script* | Facilitators discuss the next steps and experts are asked to give input | Next steps for the core modeling team |
| Evaluation of GMB process | Experts are asked to share their experiences with the online GMB process | Reflections on the GMB implementation |

*Detailed agenda*

| Time | Min | Activity | Facilitator one  *Facilitator, modeler/reflector* | Facilitator two  *Recorder, modeler/reflector* | Facilitator three  *Gatekeeper, process coach* |
| --- | --- | --- | --- | --- | --- |
| 15:55 - 16:00 | 5 | Entrance | Welcomes experts | *[Zoom, gallery view]*  Fixes technical issues  Starts recording | Welcomes experts |
| 16:00 - 16:20 | 20 | Introduction and recap + model review | Ask experts for plenary reflection on the CLD clarified in sessions three, walks the CLD through and initiates discussion on adequacy | *[Screen share, Miro]*  Shows clarified CLD  Captures outcomes on Miro board | Observes group dynamics and supports facilitation |
| 16:20 - 16:30 | 10 | Action ideas *Individual task* | Explains “action ideas” script. Gives experts +/- 10 min to pick out their most important action and decide:  1. How it connects with other factors  2. How feasible (difficult or easy to implement) and impactful it potentially is | *[Screen share, Miro]*  To show CLD and quadrant graph of impact and feasibility | Makes sure experts understand script |
| 16:30 -17:10 | 40 | Action ideas  *Group task* | Facilitator asks experts to share their actions one at a time | *[Screen share, Miro]*  Places the actions (1) in the quadrant as identified by the group and (2) in the CLD | Observes group dynamics and supports facilitation |
| 17:10 -  17:20 | 10 | Evaluation of online GMB process | Reflects back on hopes and fears script of session 1 and online GMB process | *[Zoom, gallery view]*  Asks experts  for feedback on GMB process | Observes group dynamics and supports facilitation |
| 17:20 - 17:25 | 5 | Next steps and closing | Explains what is going to happen next  Closing remarks: outlines plan for sessions five and six | *[Zoom, gallery view]*  Thanks experts | Observes group dynamics and supports facilitation  Thanks experts |

**Session Five (non-scripted facultative session)**

*Objectives*

Explicit         To clarify definitions, relations, and visuals of session four’s CLD

*Summary of activities*

| Activity | Description | Anticipated output |
| --- | --- | --- |
| Questions | Facilitators ask experts if any factors, relations, or visuals need to be altered to make the CLD more concise | Optimized CLD |

*Detailed agenda*

| Time | Min | Activity | Facilitator one  *Facilitator, modeler/reflector* | Facilitator two  *Recorder, modeler/reflector* |
| --- | --- | --- | --- | --- |
| 14:00-14:05 | 5 | Entrance | Welcomes experts | *[Zoom, gallery view]*  Fixes technical issues |
| 14:05-14:10 | 5 | Presentation of sessions goals | Elaborates on sessions’ goals and agenda | *[Zoom, gallery view]* |
| 14:10-14:55 | 45 | The questions | Asks experts in round robin fashion if any factors, relationships, or visuals need to be altered to make the CLD more concise  Asks experts if they have any suggestions how to measure the identified factors | *[Screen share, Miro]*  Shows the CLD  Asks for clarification of input |
| 14:55-15:00 | 5 | Closing | Closing remarks: explains that additions derived from this session will be added to the CLD and tested for consensus in the last session | *[Zoom, gallery view]*  Thanks experts |

**Session Six (non-scripted mandatory session)**

*Objectives*

Explicit         To establish consensus on the final CLD

To capture experts’ reflections on the online format

Implicit To close the process

*Summary of activities*

| Activity | Description | Anticipated output |
| --- | --- | --- |
| Questions | Facilitators ask experts if the CLD depicts their shared view on the problem concisely and establish consensus on the final CLD | An unambiguous consensus-based CLD |
|  |  |  |
|  |  |  |

*Detailed agenda*

| Time | Min | Activity | Facilitator one  *Facilitator, modeler/reflector* | Facilitator two  *Recorder, modeler/reflector* | Facilitator three  *Gatekeeper, process coach* |
| --- | --- | --- | --- | --- | --- |
| 20:00- 20:05 | 5 | Entrance | Welcomes experts | *[Zoom, gallery view]*  Fixes technical issues  Start recording | Welcomes experts |
| 20:05-20:07 | 2 | Welcome and introduction | Opening remarks: summarizes the process of sessions 1–5 | *[Zoom, gallery view]* | Observes group dynamics |
| 20:07 – 20:17 | 10 | Questions and establishing consensus | Asks experts as a group if they think this CLD represents their way of seeing the problem, if any adjustments are needed and if anything is unclear. | *[Share screen, Miro]*  Shows the CLD formed in session five | Observes group dynamics |
| 20:17-20:20 | 3 | Illustrating interventions | Elaborates on the effects and mechanisms of one intervention suggested in session four | *[Share screen, Miro]*  Shows the CLD formed in session five | Observes group dynamics |
| 20:20-20:25 | 5 | Capture experts’ reflections on the online format | Asks experts as a group to give feedback on the process verbally and by filling in an online questionnaire | *[Zoom, gallery view]*  Elaborates on how to fill in the online questionnaire | Observes group dynamics |
| 20:25 – 20:30 | 5 | Closing | Closing remarks: elaborates on study timeline after the sessions | *[Zoom, gallery view]*  Thanks experts | Thanks experts |

1. **Experts’ characteristics**

|  | Age | Gender | Profession/Field of expertise |
| --- | --- | --- | --- |
| D1 | 65 | F | General practitioner |
| D2 | 54 | F | District nurse |
| D3 | 46 | F | Nurse specialist geriatrics |
| D4 | 50 | M | Geriatrician |
| D5 | 55 | F | Nurse transfer coordinator |
| D6 | 43 | M | Elderly care physician |
| D7 | 46 | F | ED physician |
| D8 | 34 | M | Data analyst healthcare insurer |
| D9 | 80 | M | Patient representative |

1. **Experts’ reflections on implementation**

The experts gave feedback on the GMB process during the sessions. They reported that explanations of content, methodology, and the communication medium (Zoom and Miro) were clear and that dedicating extra time to clarifying contributing factors and interactions was important. The experts believed that the session output was clarified and the CLD was organized and visualized in a way that clearly depicted their shared view of the problem. Experts were surprised by the overview the CLD provided. Zoom and Miro were rated as good forms of communication. Experts also said that an advantage of the online format was that it allowed more time-efficient participation and fitted better to their schedule than in-persons meetings do. The main disadvantage of the online format was not being able to see other experts in person and not being able to network. Experts valued the role of the facilitators and their clear leadership during the discussions. They developed a shared enthusiasm for the process as the sessions progressed and were connected through a shared insight and belief in the methodology.

References

1. Scriptapedia. https://en.wikibooks.org/wiki/Scriptapedia (last accessed 1 June 2022).
